# Effects of hepatitis B virus infection and antiviral therapy on the clinical prognosis of nasopharyngeal carcinoma

**DOI:** 10.1002/cam4.2715

**Published:** 2019-11-27

**Authors:** Jing‐Jin Weng, Jia‐Zhang Wei, Min Li, Jin‐Long Lu, Yang‐Da Qin, He Jiang, Shen‐Hong Qu

**Affiliations:** ^1^ Department of Otolaryngology & Head and Neck The People's Hospital of Guangxi Zhuang Autonomous Region Nanning People's Republic of China

**Keywords:** antiviral therapy, hepatitis B virus, nasopharyngeal carcinoma, prognosis

## Abstract

**Purpose:**

To investigate the clinical characteristics of nasopharyngeal carcinoma (NPC) and a concomitant hepatitis B virus (HBV) infection, as well as the potential effects of HBV infection and antiviral therapy on prognosis.

**Methods:**

We conducted a retrospective chart review of all NPC patients from December 2010 to December 2014. After collecting medical records and conducting follow‐ups on patients, a total of 876 eligible NPC patients were included. For each patient, medical records were reviewed. Factors predictive of outcome were compared using the log‐rank test and Cox regression analysis.

**Results:**

Among the 876 participants, 106 (12.1%) patients were HBV‐infected patients. The hepatitis B surface antigen‐positive [HBsAg(+)] group had a lower CD^4+^ T cell count than the HBsAg(−) group (*P* = .048). Among patients with stage I/II NPC, 5‐year overall survival (OS), disease‐free survival (DFS), relapse‐free survival, and distant metastasis‐free survival (DMFS) of the HBsAg(+) group were 82.5%, 70.7%, 87.7%, and 76.6%, respectively, whereas those of the HBsAg(−) group were 91.4%, 86.0%, 93.8%, and 92.1%, respectively. Statistically significant differences in OS, DFS, and DMFS existed between both groups (*P* = .017, .018, and .004, respectively). The multivariate analysis indicated that HBsAg status and N stage are independent risk factors affecting OS, DFS, and DMFS of NPC patients. A statistically significant difference in 5‐year DMFS existed between the antivirus (90.0%) and no‐antivirus groups (70.0%) (*P* = .043).

**Conclusions:**

Hepatitis B virus infection is an independent risk factor for early stage NPC, which may be associated with its reduced immune functions compared to the HBsAg(−) group. Anti‐HBV treatment may improve the prognosis of HBV‐infected NPC patients.

## Graphical abstract

Nasopharyngeal carcinoma (NPC) is a unique head and neck cancer with a high incidence in southern China, and this area has a higher hepatitis B virus (HBV) infection rate than other regions. Hepatitis B virus infection is an independent risk factor for early‐stage NPC, which may be associated with its reduced immune functions compared to the HBsAg(−) group. Anti‐HBV treatment may improve the prognosis of HBV‐infected NPC patients.

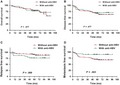

## 1 INTRODUCTION

Nasopharyngeal carcinoma (NPC) is a unique head and neck cancer that is endemic to the southern region of China.^1^ World Health Organization type III is the most common pathologic type of NPC in high‐prevalence areas and is believed to be associated with Epstein‐Barr virus (EBV) infection. The southern region of China has a higher hepatitis B virus (HBV) infection rate than other regions, with hepatitis B surface antigen‐positive [HBsAg(+)] rate of 10%‐12%.^2^ It has been previously reported that about 11% of NPC patients are infected with HBV; a further study revealed that HBV infection is an independent risk factor of locally advanced NPC.^3^ According to the National Comprehensive Cancer Network (NCCN) Clinical Practice Guidelines in Oncology, chemoradiotherapy is currently used to treat most patients with mid‐ to late‐stage NPC. Presently, the treatment failures against NPC are mainly attributed to distant metastasis. Hence, some scholars proposed the use of induction chemotherapy for patients with mid‐ to late‐stage NPC to reduce the risk of metastasis.^4^ Chemotherapy has been previously shown to induce liver injuries that led to the termination of chemotherapy in some patients, thereby affecting the continuation of treatment.^5^ Hepatitis B virus infected‐patients may undergo HBV reactivation after receiving chemotherapy.^6^ A previous study revealed that the administration of lamivudine during chemotherapy can reduce HBV reactivation and improve liver injuries.^7^ Another study found that NPC patients with active HBV had lower 5‐year relapse‐free survival (RFS) and progression‐free survival (PFS) rates than those with inactive HBV,^8^ suggesting that HBV infection may potentially affect the prognosis of NPC.

Hepatitis B virus infection is endemic in the southern region of China, implying that a considerable proportion of NPC patients are infected with HBV, or even suffering from viral hepatitis. To the best of our knowledge, no study has investigated the effects of anti‐HBV treatment on the prognosis, as well as the pretreatment and posttreatment hematologic toxicity of HBsAg(+) NPC. Our study aimed to explore the clinical characteristics of HBV‐infected NPC patients and to investigate the effects of anti‐HBV treatments on the hematologic toxicity and clinical prognosis of patients.

## 2 MATERIALS AND METHODS

### 2.1 Patient selection

Data for the patients with NPC were retrieved from the database of The People's Hospital of Guangxi Zhuang Autonomous Region (Nanning, China). This study was approved by our institutional ethical committee. All the patients or their parents provided written informed consent.

The case inclusion criteria included: (a) histopathologically diagnosed patients; (b) patients with Karnofsky score ≥80; (c) patients who have completed their radiotherapy/chemotherapy regimens; (d) patients whose informed consent has been obtained. Exclusion criteria: (a) patients with distant metastasis; (b) patients who were intolerant to radiotherapy and chemotherapy; (c) patients who terminated their treatments prematurely. A total of 876 eligible NPC patients were recruited for this study in the Nasopharyngeal Carcinoma Research Institute of the People's Hospital of Guangxi Zhuang Autonomous Region from December 2010 to December 2014. All patients were classified into different stages, in accordance with the Union for International Cancer Control (2010, 7th edition) staging system for NPC.

### 2.2 Treatment

(1) Radiotherapy: (a) two‐dimensional radiotherapy (2DRT): the accumulated radiation doses were 70‐76 Gy for primary tumor; 60‐70 Gy for positive lymph node lesions in the neck; and 50 Gy for uninvolved lymphatics in the neck. (b) Intensity modulated radiation therapy: The prescribed dose was 69‐72 Gy delivered to the PTVnx and PTVnd and 60‐65 Gy delivered to the PTV1. The PTV2 was treated to 50‐56 Gy. All patients were given one fraction daily 5 days a week.

(2) Chemotherapy: overall, 138 of 876 patients (15.8%) were treated with RT alone, and 738 of 876 patients (84.2%) received concurrent chemoradiotherapy (CCRT) or CCRT plus adjuvant chemotherapy (AC). The majority of the chemotherapy patients (718 of 738, 97.3%) received CCRT, the rest of the patients received CCRT plus AC (20 of 738, 2.7%). Two regimes of chemotherapy were frequently used: cisplatin (30 mg/m^2^ per day for 3 days) with 5‐fluorouracil (2000 mg/m^2^ for 5 days); nedaplatin (80 mg/m^2^ for 1 day) with 5‐fluorouracil (2000 mg/m^2^ for 5 days) every 4 weeks for 2‐3 cycles. Ninety‐three patients in the HBsAg(+) group (87.7%) and 625 patients in the HBsAg(−) group (81.2%) received CCRT alone. CCRT plus AT was delivered to 1 (0.9%) patient and 19 patients (2.5%) in the HBsAg(+) and HBsAg(−) groups respectively.

(3) Antiviral therapy: a total of 31 of 106 HBsAg(+) patients (29.2%) were administered with antiviral drugs as follows: 15 patients were administered with 0.5 mg of Entecavir (ETV) tablet once daily; 13 patients were administered with 100 mg of lamivudine tablet once daily; 5 patients were administered with 10 mg of adefovir tablet once daily. Those antiviral treatments were commenced 1 week before chemotherapy until 6 months after the chemotherapy.

### 2.3 HBV, EBV, and liver function tests

All patients were tested upon admission for the presence of hepatitis B surface antigen (HBsAg), hepatitis B surface antibody (HBsAb), hepatitis B e antigen, hepatitis B e antibody (HBeAb), and hepatitis B core antibody (HBcAb) via enzyme‐linked immunosorbent assay. Besides, those patients also underwent EBV‐DNA (via fluorescence‐based quantitative polymerase chain reaction [PCR]), EBV‐IgA/viral capsid antigen (VCA), EBV‐IgA/EA, and liver function test upon admission. The liver function test was repeated weekly or biweekly during hospitalization. HBsAg(+) patients also underwent HBV‐DNA tests via fluorescence‐based quantitative PCR assay. Hepatitis B virus‐DNA >500 IU/mL was considered HBV‐positive, and EBV‐DNA >0 copies/mL was considered EBV‐positive.

### 2.4 Detection of blood cells and T cell subsets

Two milliliters of venous blood was drawn before, during (at least every 2 weeks), and after treatment to measure hemoglobin (HGB), lymphocyte, neutrophil (NEUT), platelet (PLT), red blood cell, and white blood cell (WBC) counts. Those patients also underwent flow cytometric determination of T cell subsets, including the number of CD4^+^, CD8^+^, and double‐positive (DP) T cells. Liver injury and myelosuppression were assessed in accordance with the criteria for assessing toxicity and side effects of drugs in the Common Terminology Criteria for Adverse Events (Version 4.03) issued by the US National Cancer Institute.

### 2.5 Follow‐ups and endpoints

After they were discharged from the hospital, patients in both groups returned to the hospital or clinic for follow‐ups quarterly in the first year, triannually to biannually in the second year, and annually from the third year onwards. Patients who did not return to the hospital as scheduled were followed up via telephone conversations. The follow‐ups were terminated on 31 December 2018. The shortest follow‐up duration was 26 months, whereas the longest follow‐up duration was 99 months with a median follow‐up duration of 65 months. Distant metastasis‐free survival (DMFS) was defined as the time from randomization until the date of first distant metastasis or death from any cause. The following end‐points were assessed: overall survival (OS), disease‐free survival (DFS), RFS and DMFS. The OS was defined as the time from the diagnosis of NPC to death from any cause or until the date of the last follow‐up. DFS was defined as the time from diagnosis to events that included death or disease progression (local, regional, or distant metastasis) or until the date of the last follow‐up. Relapse‐free survival was defined as the time from diagnosis until the date of first recurrence (local, regional or distant metastasis) or until the date of the last follow‐up. Distant metastasis‐free survival was defined as the time from diagnosis until the date of first distant metastasis or until the date of the last follow‐up.

### 2.6 Statistical analysis

Statistical analyses were carried out using the SPSS 24.0 statistical software. The count data were compared between the two groups using the chi‐square (*χ* ^2^) test. Normally distributed data were compared using *t* test or ANOVA, whereas non‐normally distributed data were compared using the rank‐sum test. Univariate survival analysis was performed using Kaplan‐Meier survival curves, which were compared between groups via the log‐rank test. Multivariate analysis was performed using the Cox proportional hazards model. *P* < .05 indicated the presence of statistically significant differences.

## 3 RESULTS

### 3.1 Patient characteristics

In total, 106 of the 876 patients (12.1%) were positive for HBsAg. No significant differences in gender, stage, therapeutic methods, and RT techniques existed between the two groups (*P* > .05), but the HBsAg(+) group had a higher proportion of patients aged ≤50 years than the HBsAg(−) group (Table 1).

**Table 1:** Baseline characteristics of patients

Characteristics	Number (percentage) of patients (n = 876)	HBsAg(−) group (n = 770)	HBsAg(+) group (n = 106)	*χ* ^2^	*P*‐value
Gender					
Male	624 (71.2)	543 (70.5)	81 (76.4)	1.58	.209
Female	252 (28.8)	227 (29.5)	25 (23.6)		
Age					
≤50 y	466 (53.2)	397 (51.6)	69 (65.1)	6.857	**.009**
>50 y	410 (46.8)	373 (48.4)	37 (34.9)		
T stage					
1	93 (10.6)	78 (10.1)	15 (14.2)	1.965	.58
2	296 (33.8)	263 (34.2)	33 (31.1)		
3	251 (28.7)	223 (28.9)	28 (26.4)		
4	236 (26.9)	206 (26.8)	30 (28.3)		
N stage					
0	267 (30.5)	231 (30)	36 (34)	1.721	.632
1	310 (35.4)	274 (35.6)	36 (34)		
2	224 (25.6)	201 (26.1)	23 (21.7)		
3	75 (8.6)	64 (8.3)	11 (10.4)		
Clinical stage					
I	42 (4.8)	36 (4.7)	6 (5.7)	2.629	.452
II	227 (25.9)	198 (25.7)	29 (27.4)		
III	308 (35.2)	278 (36.1)	30 (28.3)		
IV	299 (34.1)	258 (33.5)	41 (38.7)		
Treatment					
RT alone	138 (15.8)	126 (16.4)	12 (11.3)	1.785	.181
CRT	738 (84.2)	644 (83.6)	94 (88.7)		
Radiotherapy					
IMRT	538 (61.4)	472 (61.3)	66 (62.3)	0.037	.848
2DRT	338 (38.6)	298 (38.7)	40 (37.7)		

Abbreviations*:* 2DRT, 2‐dimensional radiotherapy; CRT, Chemoradiotherapy; IMRT, intensity modulated radiotherapy; RT, radiotherapy.

### 3.2 Comparison of T cell subsets between HBsAg(+) and HBsAg(−) group

The HBsAg(+) group had a lower CD4^+^ T cell count than the HBsAg(−) group (*P* = .048). No significant differences in the CD8^+^ T cell count, CD4^+^/CD8^+^ ratio, and DP (CD4^+^ and CD8^+^) T cell (DP‐T cell) count existed between the two groups (*P* > .05) (Table 2).

**Table 2:** Comparison of T‐cell subsets between the two groups

T‐cell subsets	HBsAg(‐) group	HBsAg(+) group	*t*	*P*‐value
CD4^+^ T cells	53.76 ± 10.59	49.41 ± 10.51	1.988	**.048**
CD8^+^ T cells	42.1 ± 10.22	43.82 ± 8.46	−0.827	.409
DP‐T cells	1.23 ± 1.20	1.17 ± 1.11	0.242	.809
CD4^+^/CD8^+^ ratio	1.43 ± 0.68	1.21 ± 0.48	1.588	.114

Abbreviation: CD4^+^ and CD8^+^, double‐positive T cells.

### 3.3 Survival analysis of study participants

**Figure 1:**
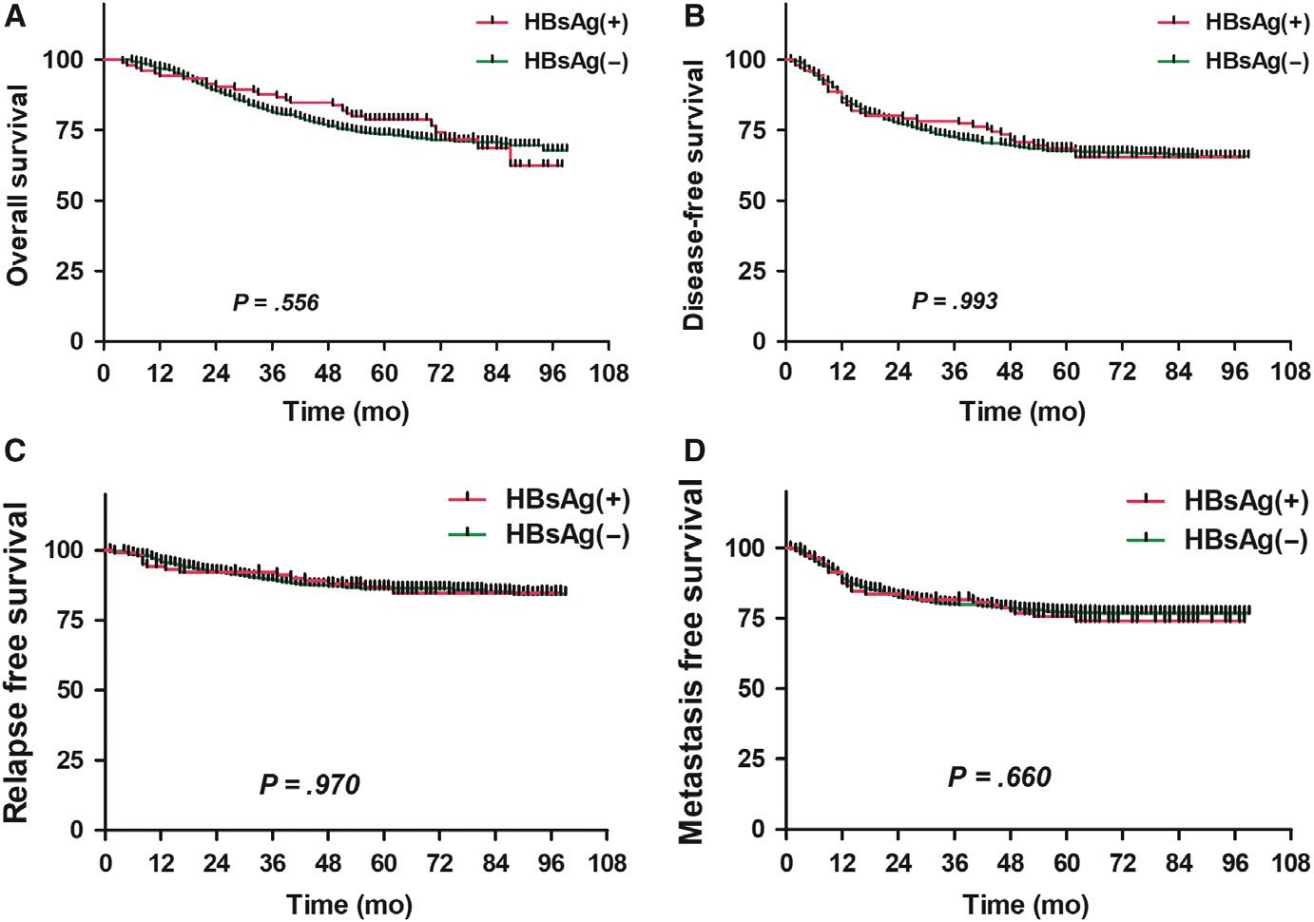
Comparison of survival curves between hepatitis B surface antigen (HBsAg)‐positive and HBsAg‐negative patients with nasopharyngeal carcinoma (A, overall survival; B, disease‐free survival; C, relapse‐free survival; D, metastasis‐free survival)

No statistically significant differences in 5‐year OS, DFS, RFS, and DMFS existed between the HBsAg(+) group (106 patients; 78.4%, 68.1%, 86.6%, and 75.5%, respectively) and the HBsAg(−) group (770 patients; 73.5%, 67.5%, 86.1%, and 77.2%, respectively) (*P* > .05) (Figure 1). The multivariate analysis revealed that age, T stage, N stage, clinical stage, therapeutic method, and RT techniques are independent risk factors affecting OS of NPC patients; age, T stage, N stage, and therapeutic method are independent risk factors affecting the DFS of NPC patients; clinical stage and RT techniques are independent risk factors affecting the RFS of NPC patients; age, N stage, clinical stage, and therapeutic method are independent risk factors affecting the DMFS of NPC patients (*P* < .05) (Table 3).

**Table 3:** Multivariate analysis of prognostic factors affecting NPC patients (n = 876)

Factor	OS		DFS		RFS		DMFS	
	HR	*P*‐value	HR	*P*‐value	HR	*P*‐value	HR	*P*‐value
Age	1.637	**<.001**	1.476	**.001**	NA	NA	1.389	**.023**
T stage	1.319	**.028**	1.322	**.017**	NA	NA	NA	NA
N stage	1.327	**<.001**	1.322	**<.001**	NA	NA	1.282	**.001**
Clinical stage	1.393	**.041**	1.313	.069	1.871	**<.001**	1.849	**<.001**
Chemotherapy	0.612	**.001**	0.66	**.004**	NA	NA	0.638	**.008**
RT techniques	1.337	**.029**	NA	NA	1.529	**.024**	NA	NA

Abbreviations: DFS, disease‐free survival; DMFS, distant metastasis‐free survival; HR, hazard ratio; NPC, nasopharyngeal carcinoma; OS, overall survival; RFS, relapse‐free survival; RT, radiotherapy.

### 3.4 Effects of PLT count on prognosis

**Figure 2:**
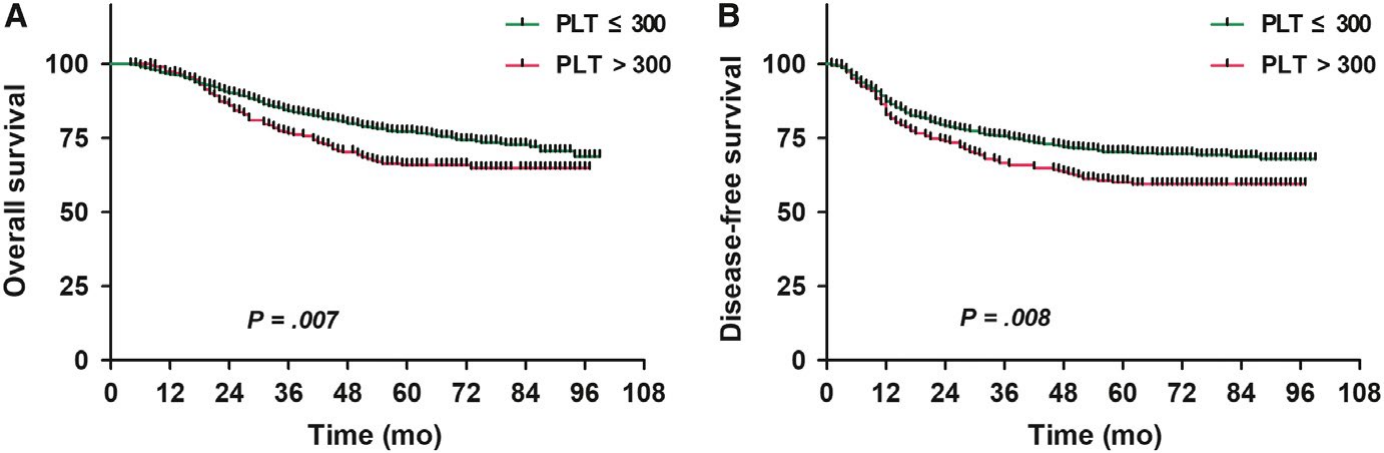
Comparison of survival curves between normal platelets and high platelets patients with nasopharyngeal carcinoma (A, overall survival; B, disease‐free survival)

The high PLT group (PLT > 300 × 10^9^ cells/L) had lower 5‐year OS and DFS than the normal PLT group (PLT ≤ 300 × 10^9^ cells/L) (OS: 65.6% vs 76.9%, *P* = .007; DFS: 59.8% vs 70.2%, *P* = .008) (Figure 2).

### 3.5 Effects of HBV infection on the survival rate of patients with early stage and locally advanced NPC

**Figure 3:**
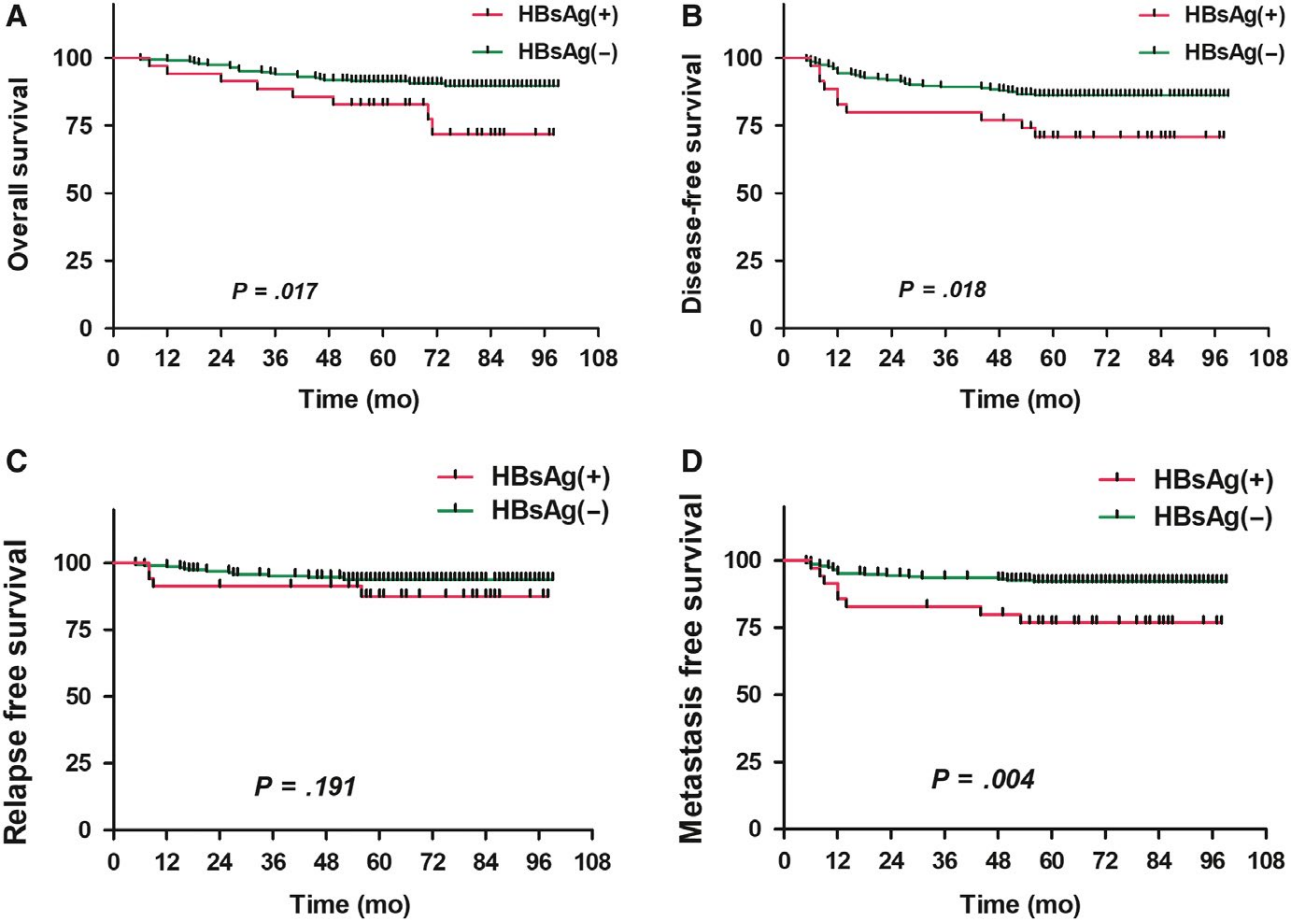
Comparison of survival curves between hepatitis B surface antigen (HBsAg)‐positive and HBsAg‐negative patients with early‐stage nasopharyngeal carcinoma (A, overall survival; B, disease‐free survival; C, relapse‐free survival; D, metastasis‐free survival)

Our subgroup analysis on patients with different stages of NPC revealed that among patients with stage I/II NPC, the 5‐year OS. DFS, RFS, and DMFS of the HBsAg(+) group (35 patients) were 82.5%, 70.7%, 87.7%, and 76.6%, whereas those of the HBsAg(−) group (234 patients) were 91.4%, 86.0%, 93.8%, and 92.1% respectively. Statistically significant differences in OS, DFS, and DMFS existed between the two groups (*P* = .017, .018, and .004, respectively), but no significant difference in RFS existed between the two groups (*P* = .191) (Figure 3). Multivariate analysis also revealed that HBsAg status and N stage were independent risk factors affecting OS, DFS, and DMFS of NPC patients (Table 4). Among patients with stage III/IV NPC, no significant differences in 5‐year OS, DFS, RFS, and DMFS existed between the HBsAg(+) group (71 patients; 76.3%, 66.8%, 86.0%, and 75.0%, respectively) and the HBsAg(−) group (536 patients; 65.7%, 59.4%, 82.5%, and 70.6%, respectively) (all *P* > .05). Of the 106 HBsAg(+) patients, 18 were at immune‐tolerant phase, 7 were at immune‐active phase, and 81 were at inactive chronic HBV. Our subgroup analysis on patients with different phases of chronic HBV infection revealed that no significant difference in OS, DFS, RFS, and DMFS between the three groups (*P* = .998, .787, .571, and .412 respectively).

**Table 4:** Multivariate analysis of prognostic factors affecting patients with early‐stage NPC (n = 269)

Factor	OS		DFS		RFS		DMFS	
	HR	*P*‐value	HR	*P*‐value	HR	*P*‐value	HR	*P*‐value
HBsAg status	2.843	**.012**	2.528	**.011**	NA	NA	3.689	**.002**
T stage	NA	NA	NA	NA	NA	NA	NA	NA
N stage	2.775	**.014**	2.627	**.005**	NA	NA	4.533	**.002**
Clinical stage	NA	NA	NA	NA	NA	NA	NA	NA

Abbreviations: DFS, Disease‐free survival; DMFS, distant metastasis‐free survival; HR, hazards ratio; NPC, nasopharyngeal carcinoma; OS, overall survival; RFS, relapse‐free survival.

### 3.6 Relationship between EBV and HBV infections, and their relationship with prognosis

**Figure 4:**
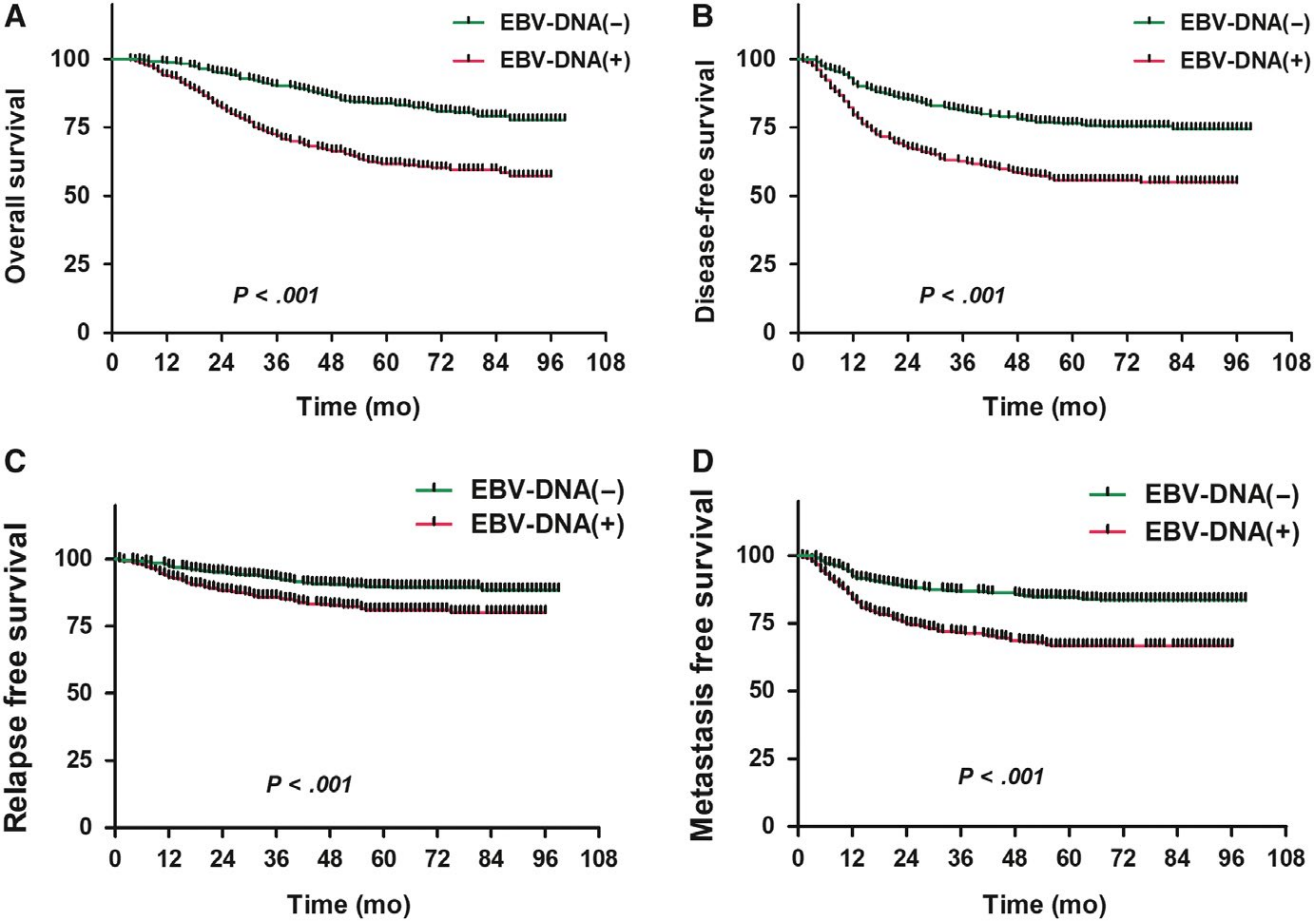
Comparison of survival curves between Epstein‐Barr (EBV)‐DNA‐positive and EBV‐DNA‐negative patients with nasopharyngeal carcinoma (A, overall survival; B, disease‐free survival; C, relapse‐free survival; D, metastasis‐free survival)

**Figure 5:**
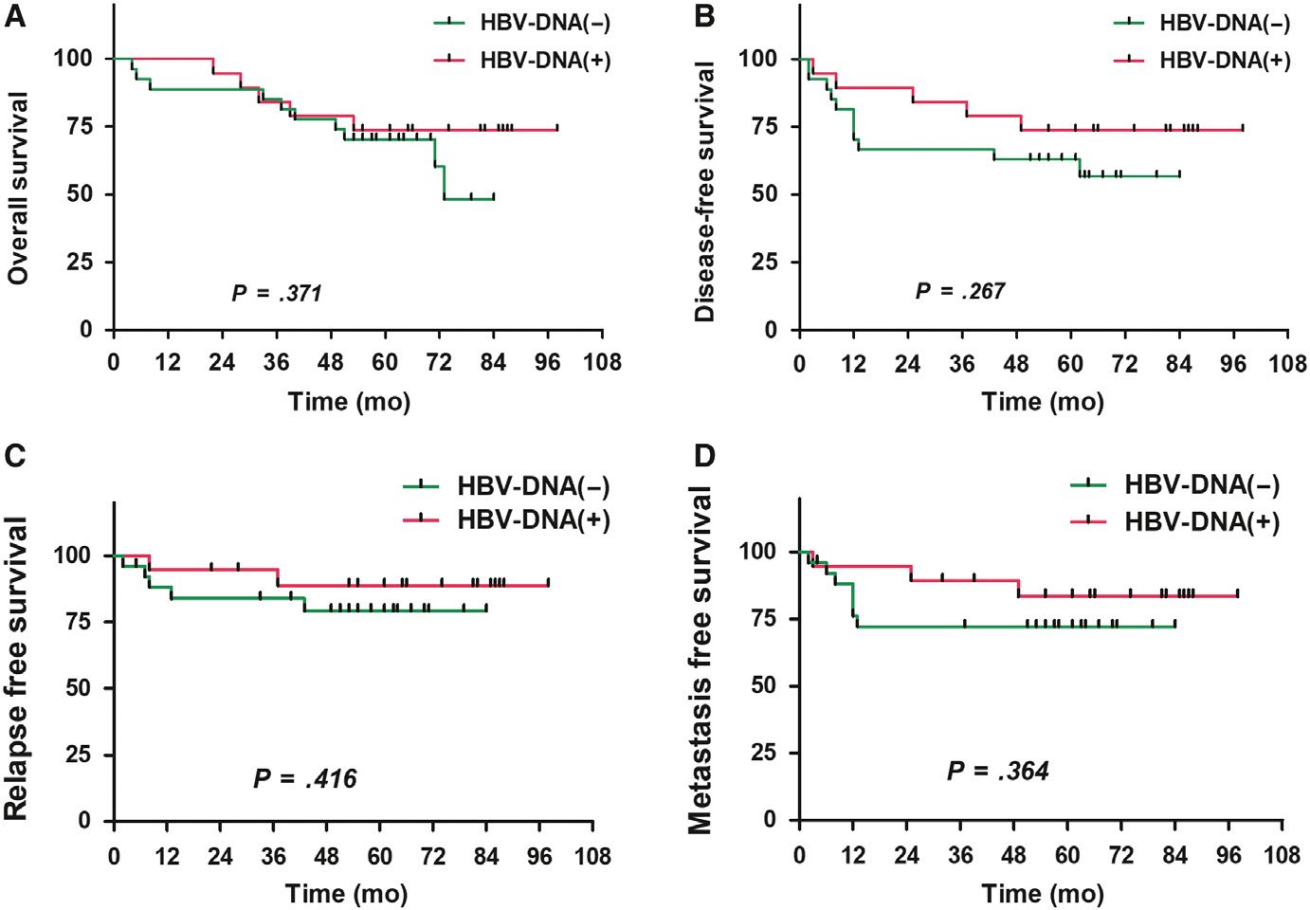
Comparison of survival curves between hepatitis B virus (HBV)‐DNA‐positive and HBV‐DNA‐negative patients with nasopharyngeal carcinoma (A, overall survival; B, disease‐free survival; C, relapse‐free survival; D, metastasis‐free survival)

The median of EBV in the total cohort was 0 copies/mL (range, 0~4.0 × 10^6^ copies/mL), and the median of HBV in HBsAg(+) group was 0 IU/mL (range, 0~5.2 × 10^6^ IU/mL). Before treatment: no significant correlation existed between EBV‐DNA and HBV‐DNA (rs = −0.155, *P* = .315); EBV‐DNA(−) patients had better 5‐year OS, DFS, RFS, and DMFS than EBV‐DNA(+) patients (OS: 83.5% vs 61.6%, *P* < .001; DFS: 76.3% vs 55.5%, *P* < .001; RFS: 89.8% vs 80.8%, *P* < .001; DMFS: 84.6% vs 66.6%, *P* < .001) (Figure 4). In addition, the EA‐IgA(−) group had a better OS than the EA‐IgA(+) group (*P* = .037), but no significant differences in DFS, RFS, and DMFS existed between the two groups (*P* > .05). Additionally, no significant differences in OS, DFS, RFS, and DMFS existed between the VCA‐IgA(−) group and the VCA‐IgA(+) group (*P* > .05). Patients in both HBV‐DNA(−) and HBV‐DNA(+) groups had similar 5‐year OS, DFS, RFS, and DMFS (OS: 68.9% vs 73.5%, *P* = .371; DFS: 63.0% vs 73.5%, *P* = .267; RFS: 80.1% vs 88.8%, *P* = .416; DMFS: 73.3% vs 83.2%, *P* = .364) (Figure 5). Besides, no significant differences in OS, DFS, RFS, and DMFS existed between positive and negative groups of HBcAb, HBeAb, and HBsAb (ALL *P* > .05).

### 3.7 Effects of anti‐HBV treatments on prognosis

**Figure 6:**
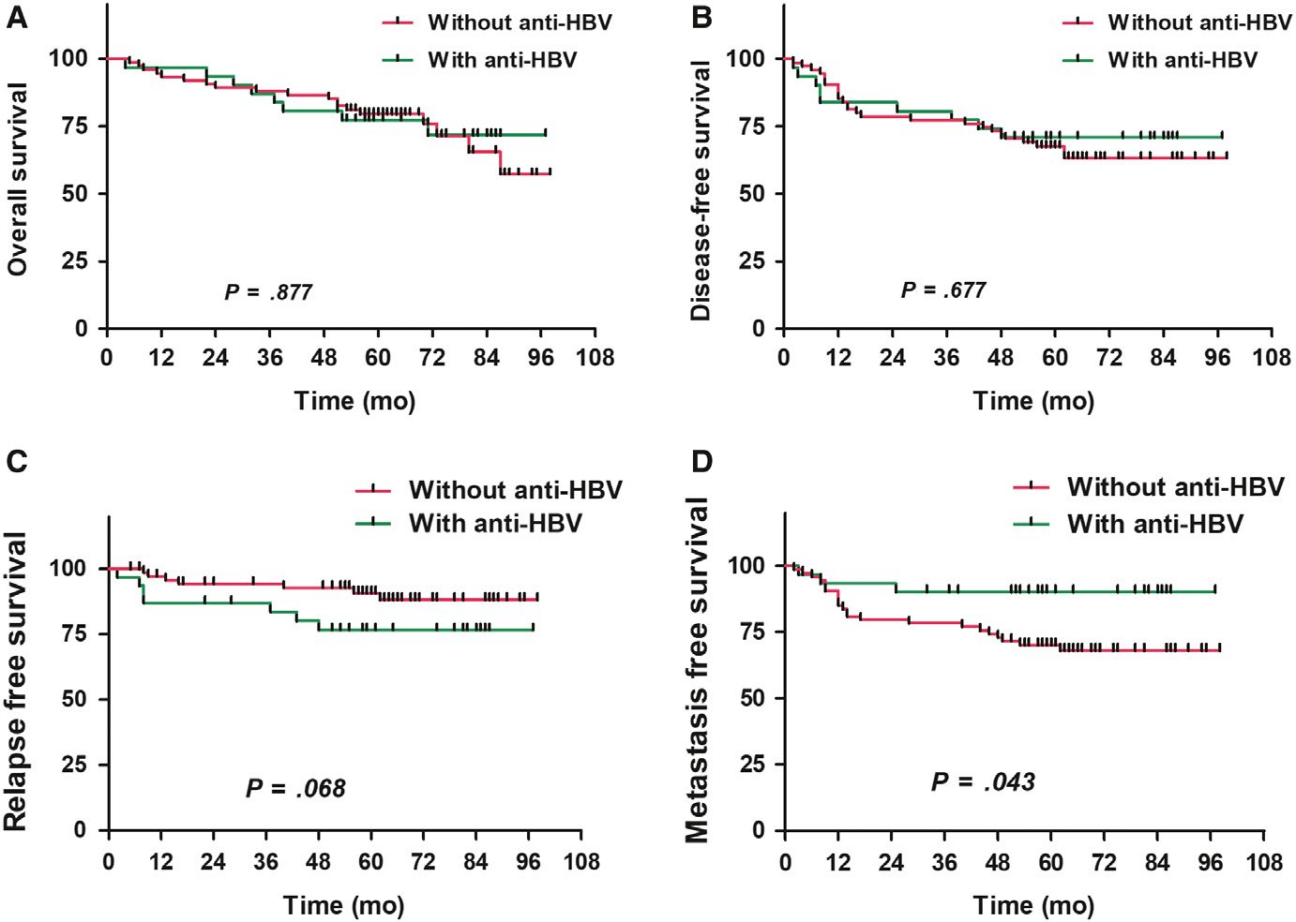
Comparison of survival curves between nasopharyngeal carcinoma patients with and without anti‐hepatitis B virus (HBV) treatment (A, overall survival; B, disease‐free survival; C, relapse‐free survival; D, metastasis‐free survival)

Of the 106 HBsAg(+) patients, 31 patients received antiviral therapy, whereas the remaining 75 patients received no antiviral therapy. The 5‐year OS, DFS, RFS, and DMFS of the antivirus group were 76.9%, 70.5%, 76.1%, and 90.0%, whereas those of the no‐antivirus group were 79.0%, 67.1%, 91.1%, and 70.0% respectively. A significant difference in DMFS existed between the two groups (*P* = .043), but not in their OS, DFS, and RFS (*P* = .877, .677, and .068) (Figure 6).

### 3.8 Effects of anti‐HBV treatments on liver function, blood cells, and EBV‐DNA

No significant difference in the incidence rate of grade III‐IV hematologic toxicity existed between the HBsAg(+) and HBsAg(−) groups during treatment (Table 5). Before treatment, 5 patients had grade 1 liver injuries in the no‐antivirus group, but none had grade 3 liver injury. On the other hand, 6 patients and 1 patient had grade 1 and 3 liver injuries in the antivirus group, respectively (*P* = .040). During treatment, 9 patients had grade 1 liver injuries, whereas 1 patient had grade 2, 3, and 4 liver injuries each in the no‐antivirus group. On the other hand, 5 patients had grade 1 liver injuries, whereas none had liver injuries higher than grade 1 in the antivirus group; however, no statistically significant difference existed between the two groups (*P* > .05). At the end of treatment, 4 patients still had liver injuries in the no‐antivirus group, whereas the liver function parameters of patients in the antivirus group were normal. During treatment, 9, 5, and 1 patient had grade 1, 2, and 3 PLT count declines in the antivirus group, respectively; whereas 7, 8, and 2 patients had grade 1, 2, and 3 PLT count declines in the no‐antivirus group (*P* = .041) respectively. No significant differences in HGB, WBC count decline, and NEUT count decline existed between both groups (*P* > .05) (Table 6). Before treatment, 16 and 32 EBV‐DNA(+) patients were in the antivirus group and the no‐antivirus group respectively. At the end of treatment, only 1 and 3 EBV‐DNA(+) patients remained in the antivirus and no‐antivirus groups respectively.

**Table 5:** Hematologic toxicity in the HBsAg(−) and HBsAg(+) groups during treatment

Hematologic toxicity	HBsAg(−) group (n = 770) (%)	HBsAg(+) group (n = 106) (%)	*χ* ^2^	*P*‐value
Anemia				
Grade 0‐2	744 (96.62)	100 (94.34)	0.808	.369
Grade 3‐4	26 (3.38)	6 (5.66)		
Leukopenia				
Grade 0‐2	694 (90.13)	90(84.91)	2.705	.100
Grade 3‐4	746(9.87)	16(15.09)		
Neutropenia				
Grade 0‐2	726 (94.29)	96 (90.57)	2.229	.135
Grade 3‐4	44 (5.71)	10 (9.43)		
Thrombocytopenia				
Grade 0‐2	747 (97.01)	103 (97.17)	0.000	1.000
Grade 3‐4	23 (2.99)	3 (2.83)		

**Table 6:** Hematologic toxicity in the antivirus and no‐antivirus groups during treatment

Hematologic toxicity	No‐antivirus group (n = 75) (%)	Antivirus group (n = 31) (%)	*χ* ^2^	*P*‐value
Anemia				
Grade 0	45 (60)	14 (45.16)	1.957	.162
Grade 1‐4	30 (40)	17 (54.84)		
Leukopenia				
Grade 0	11 (14.67)	4 (12.9)	0.000	1.000
Grade 1‐4	64 (85.33)	27 (87.1)		
Neutropenia				
Grade 0	32 (42.67)	9 (29.03)	1.719	.190
Grade 1‐4	43 (57.33)	22 (70.97)		
Thrombocytopenia				
Grade 0	58 (77.33)	16 (51.61)	6.885	**.009**
Grade 1‐4	17 (22.67)	15 (48.39)		

## 4 DISCUSSION

In this study, we found that HBV infection affects the prognosis of patients with early‐stage NPC; HBV‐infected NPC patients who received antiviral therapy outperformed those who did not receive antiviral therapy in DMFS. A previous study reported that about 11% of NPC patients are also infected by HBV.^3^ Our study also yielded similar results, where HBV‐infected patients accounted for 12.1% of all NPC patients included in this study. Our literature search revealed that currently two studies^3^, ^8^ have reported the effects of HBV infection on the prognosis of NPC. Both studies suggested that HBsAg(+) patients have a poorer prognosis than HBsAg(−) patients. In this study, we found that no significant difference in survival rates existed between HBsAg(+) NPC and HBsAg(−) NPC patients, but the former displayed lower OS, DFS, and DMFS than the latter among those with early‐stage NPC. Liu et al^3^ inferred that the effects of HBV infection on the prognosis of NPC may be associated with its impacts on the host immune functions, but they did not report the differences in immune functions between the two groups of patients. Recently, a study revealed that patients with chronic Hepatitis B and cirrhosis had lower DP‐T, CD4^+^ T, and CD8^+^ T cell counts than the normal population.^9^ Our comparison of T cell subsets between the two groups also revealed that the HBsAg(+) group had a lower CD4^+^ T cell count than the HBsAg(−) group. However, we did not observe any significant difference in the CD8^+^ T cell count between the two groups. Another study also revealed that the decline in CD4^+^ T cell count can promote the onset of liver cancer.^10^ According to the NCCN Clinical Practice Guidelines in Oncology, chemoradiotherapy is preferred for mid‐ to late‐stage NPC, whereas radiotherapy alone is sufficient for patients with early‐stage NPC.^11^ Some scholars inferred that the poorer prognosis among patients with early‐stage NPC in the HBsAg(+) group than those in the HBsAg(−) group may be attributed to increased inflammation and increased carcinogenicity during co‐infection with two viruses.^12^ A more aggressive treatment, such as chemotherapy, is usually required for patients with increased malignancy. However, patients with early‐stage NPC have an increased risk of metastasis as they did not receive systemic chemotherapy, as relatively few patients received chemotherapy. In contrast, most patients with mid‐ to late‐stage NPC received synchronous chemotherapy, which may have reduced the risk of distant metastasis after treatment.

World Health Organization type III is the most common pathologic type of NPC, and is generally believed to be associated with EBV infection. Numerous studies have demonstrated that EBV‐DNA is associated with the recurrence, metastasis, and prognosis of tumors.^13^, ^14^ Our study also yielded similar conclusions. Besides, our results also revealed that the EA‐IgA(−) group had a better OS than the EA‐IgA(+) group before treatment, indicating that EBV is associated with the prognosis of NPC. Our further studies discovered that no correlation existed between high HBV‐DNA and NPC prognosis. Currently, a paper has reported the absence of a significant correlation between EBV infection and HBV infection.^8^ Our study also yielded similar conclusions, suggesting that EBV and HBV have different effects on NPC, and probably affect the onset and progression of NPC via different pathways. Some scholars speculated that the former promotes the progression of tumors, whereas the latter affects the tumor microenvironment.^8^ In addition, some scholars believe that the two viruses co‐infecting the same host indirectly affect each other via the host immune system rather than via direct interaction with each other.^15^ A previous study revealed that administration of an anti‐HBV drug, lamivudine, to NPC patients receiving chemotherapy, can reduce the incidences of HBV reactivation and chemotherapy‐related hepatitis.^7^ Studies on other types of tumors also revealed that antiviral therapy can reduce the incidence of HBV reactivation.^16^, ^17^ Our results showed that the no‐antivirus group had 3 patients with liver injuries higher than grade 1, whereas no patient had liver injuries higher than grade 1 in the antivirus group; however, no statistically significant difference existed between the two groups. Four patients still had liver injuries in the no‐antivirus group at the end of treatment, whereas liver function parameters of patients in the antivirus group were normal, indicating that antiviral therapy can help reduce liver injuries.

In this study, the 5‐year DMFS of the antivirus and no‐antivirus groups were 90.0% and 70.0%, respectively, suggesting that antiviral therapy can help reduce the incidence of distant metastasis. Currently, relatively few papers have reported the effects of antiviral therapy on tumors. Some studies on liver cancer revealed that antiviral therapy can reduce the recurrence rate of liver cancer^18^ and improve the prognosis of liver cancer to a certain extent.^19^ However, another study found that antiviral drugs did not improve the prognosis of patients with liver cancer.^20^ We conducted routine blood tests on 106 HBsAg(+) NPC patients, and the results revealed that 48.4% of patients (15/31) in the antivirus group had declined PLT counts during treatment, whereas only 22.7% of patients (17/75) in the no‐antivirus group had declined PLT counts, suggesting that antiviral therapy may affect the PLT count in patients. Current studies on various types of tumors found that patients with increased PLT counts have poor prognosis.^21^, ^22^ Our univariate analysis also demonstrated that NPC patients with high PLT counts (>300 × 10^9^ cells/L) had a poorer prognosis than those with normal PLT counts. A previous study found that PLT‐derived transforming growth factor‐β (TGF‐β) can activate TGF‐β/Smad and nuclear factor kappa‐B pathways in cancer cells, thereby promoting the epithelial‐mesenchymal transition of tumor cells.^23^ Antiplatelet drugs, such as aspirin, can improve to some extent the prognosis of patients with head and neck cancers.^24^ Although ETV and lamivudine have been reported to be capable of reducing PLT count,^25^, ^26^ their underlying mechanisms of action remain unclear. Some scholars believe that the reduction in PLT counts by ETV and lamivudine is associated with cytokine secretion and immunomodulation.^27^ Besides, a study reported that for patients co‐infected with two viruses, the antiviral therapy targeting one of the viruses will increase the replication of the other virus.^28^ Although 31 HBsAg(+) NPC patients underwent anti‐HBV treatment, one EBV‐DNA(+) patient remained after treatment, indicating that anti‐HBV treatment does not increase the replication of EBV‐DNA.

Our study has several limitations. Firstly, this is a retrospective study, in which EBV‐DNA and HBV‐DNA were not assayed for some of those 876 NPC patients. Secondly, our study is similar to other studies,^3^, ^8^ whereby patients in the HBsAg(+) group tend to be younger than those in the HBsAg(−) group. Lastly, few patients received antiviral therapy (only 31 patients). Hence, our results still need to be validated with a larger sample size.

Our study suggested that patients with both HBV infection and early‐stage NPC have a poorer clinical prognosis than patients without HBV infection, which may be associated with their weaker immune system functions. The concomitant chemoradiotherapy and anti‐HBV treatment may help improve the prognosis of tumors for this type of patient. However, the exact mechanisms by which HBV affects the prognosis of NPC and anti‐HBV treatment improves the prognosis still need to be clarified in the future.

## CONFLICT OF INTEREST

The authors made no disclosures.

## Data Availability

The datasets used and/or analyzed during this study are available from the corresponding author on reasonable request.
